# South African Family Practice Manual (third edition): Book Review

**DOI:** 10.4102/phcfm.v7i1.882

**Published:** 2015-06-24

**Authors:** Bernard Gaede

**Affiliations:** 1 Department of Family Medicine, University of KwaZulu-Natal, South Africa

The third edition of the *South African Family Practice Manual* has recently been published. Over the years, I used both previous editions extensively. The first edition was a collection of articles that had appeared in *South African Family Practice* (under the guidance of Manfred Teichler), focusing on common practical aspects of family practice. The second edition of the manual was a substantive rework of many of the topics.

The third edition of this valuable manual is timely, as there have been a number of policy developments in South Africa that impact significantly on how family medicine and generalist care are conceived and practised. The content of the manual covers skills and procedures across a wide spectrum, from primary care activities in the community to more procedural skills of the hospital generalist. A wide range of approaches and activities commonly encountered in generalist medicine in the South African context is included.

The change in the approach to chapter titles in the current edition is noteworthy. In the previous (second) edition, the chapter title announced the topic, for example ’Growth monitoring’. In the new edition, however, chapter titles focus more explicitly on the practical aspects of the topic, for example ’How to do growth monitoring’. It is clear that the current edition is concerned with guiding the reader in getting things done rather than focusing on imparting theoretical knowledge or serving as background reading on any of the topics covered. The focus is clearly on practical application.

The 178 (!) chapters are organised into 15 sections, each of a particular focus area (e.g. child health or consultation). Although many, the chapters are short – generally two or three pages – and focus on key aspects of the procedure or topic. The chapter on interpreting electrocardiograms (ECGs) is longer, though. A number of new sections, such as the one on anaesthetics and another addressing community-level care and working with ward-based outreach teams, in particular, expand the scope of the manual.

The manual remains an invaluable source of easily accessible, useful lists, tables and graphs. Many of the resources are in a format that can be easily photocopied and displayed in the consulting room. However, care needs to be taken to ensure that the approaches align with local service standards and provincial or national standards such as the Essential Drug List and Standard Treatment Guidelines.

Many of the chapters presuppose a basic level of knowledge and skill, particularly with regard to high-risk knowledge areas such as anaesthetics and obstetrics. It would be advised that readers who are not familiar with the specific skills supplement their use of the manual with guidance or supervision from a more experienced doctor in order to apply the guidelines safely. A section on how to develop new skills and how to ask for help – relevant to all doctors – may be a very valuable addition to future editions.

Although the manual clearly cannot cover every procedure or skill relevant to district hospitals, there are notable gaps. In the section on surgery, fewer procedures are covered than what may generally be performed at district hospital level, such as skin grafts, dealing with hand sepsis or amputations. Similarly, in the section on consultation and working in the larger system, reference to cross-cultural consultations and how to do outreach visits will be important additions to the manual. A focus on implementing a more comprehensive chronic care model will also become increasingly important considering the changing burden of disease. With reference to the trend in policy developments, the editors may have to consider an extension on how to work in the community in the next edition, including using data for supervision of teams, how to engage with communities and the engagement of private providers and traditional healers.

Despite the mentioned shortcomings, the manual is an invaluable resource for all doctors – from junior clinicians who need to learn new skills to senior colleagues who want to refresh their memory when performing a procedure they have not done for a while. At the level of a team of doctors, the stepwise approach of the manual can be used to develop local standards of care or auditing services as part of a clinical governance process. The manual is therefore important to both junior and senior doctors working in a setting where generalists have a wide scope of practice. As such it will have great relevance in many settings throughout Africa.

